# Droplet-based digital antibiotic susceptibility screen reveals single-cell clonal heteroresistance in an isogenic bacterial population

**DOI:** 10.1038/s41598-020-60381-z

**Published:** 2020-02-24

**Authors:** Ott Scheler, Karol Makuch, Pawel R. Debski, Michal Horka, Artur Ruszczak, Natalia Pacocha, Krzysztof Sozański, Olli-Pekka Smolander, Witold Postek, Piotr Garstecki

**Affiliations:** 1 Institute of Physical Chemistry, Polish Academy of Sciences, Kasprzaka 44/52, 01-224 Warsaw, Poland; 2 Department of Chemistry and Biotechnology, Tallinn University of Technology, Akadeemia tee 15, 12618 Tallinn, Estonia; 3 Institute of Molecular and Cell Biology, University of Tartu, Riia 23, 51010 Tartu, Estonia; 4 Division of Chemistry and Chemical Engineering, California Institute of Technology, Pasadena, California 91125 USA

**Keywords:** Antimicrobials, Microfluidics

## Abstract

Since antibiotic resistance is a major threat to global health, recent observations that the traditional test of minimum inhibitory concentration (MIC) is not informative enough to guide effective antibiotic treatment are alarming. Bacterial heteroresistance, in which seemingly susceptible isogenic bacterial populations contain resistant sub-populations, underlies much of this challenge. To close this gap, here we developed a droplet-based digital MIC screen that constitutes a practical analytical platform for quantifying the single-cell distribution of phenotypic responses to antibiotics, as well as for measuring inoculum effect with high accuracy. We found that antibiotic efficacy is determined by the amount of antibiotic used per bacterial colony forming unit (CFU), not by the absolute antibiotic concentration, as shown by the treatment of beta-lactamase-carrying *Escherichia coli* with cefotaxime. We also noted that cells exhibited a pronounced clustering phenotype when exposed to near-inhibitory amounts of cefotaxime. Overall, our method facilitates research into the interplay between heteroresistance and antibiotic efficacy, as well as research into the origin and stimulation of heterogeneity by exposure to antibiotics. Due to the absolute bacteria quantification in this digital assay, our method provides a platform for developing reference MIC assays that are robust against inoculum-density variations.

## Introduction

Antibiotic resistance is one of the largest threats to global health^1,2^. In 2015, >650,000 cases of antibiotic-resistant infections were reported in the EU/EEA area, of which >30,000 resulted in death^3^. In the same year, the World Health Organization endorsed a global action plan to tackle this challenge, including calling for better understanding of the mechanisms underlying resistance and for developing better diagnostic tools^4^.

Often resistance arises against beta-lactam antibiotics^3^, which target cell wall-synthesis mechanisms in bacteria^5,6^. In Gram-negative bacteria, beta-lactamase enzymes that degrade beta-lactam antibiotics often cause the resistance. Genes encoding beta-lactamases evolve quickly and spread horizontally, driving the spread of resistance^5^. One of the most widespread beta-lactamase protein families associated with resistance is the TEM family^5,7^.

Resistance often evolves when even only a few bacteria escape antibiotic treatment. While the genetic mechanisms of drug resistance (mutations, plasmid transfer, etc.) are quite well known^8,9^, associated phenotypic drivers (such as phenotypic heterogeneity) remain poorly studied. Various mechanisms underlie phenotypic heterogeneity^10,11^; different protein-expression levels can confer ‘resistance’ on some fraction of the bacteria^12^, and persistence can arise when some fraction of bacteria survive by remaining dormant during antibiotic treatment^11^.

Heteroresistance is a form of phenotypic heterogeneity in which seemingly susceptible isogenic bacterial populations contain resistant sub-populations^13^. It is widely prevalent in pathogenic bacteria^14^, causing possible false negatives in antibiotic susceptibility testing^14,15^. Thus, there is an urgent need for efficient methods for characterizing heteroresistance, which currently can only be approximated with methods that are labour- and time-intensive^14^.

Here, we tackled this challenge with a strategy reliant on droplet microfluidics: micro-channels are used to disperse aqueous samples into an oil phase in a controlled fashion, yielding thousands of parallel reaction vessels^16–18^. Droplets enable single-cell encapsulation on a massive scale, opening avenues to high-throughput analysis of bacteria and their responses to antibiotics at the single-cell level^19^. We developed a method for droplet-based digital quantification of the distribution of clonal heteroresistance at the single-cell level, and we integrated a digital susceptibility assay against cell density. We demonstrated that, for the antibiotic-bacteria combination tested here, it is the amount of antibiotic per bacterium and not the antibiotic concentration per se that determines growth inhibition. Our approach also uncovered and quantified the tendency of bacteria to agglomerate at near-inhibitory drug conditions, highlighting that this method could be important for research into the onset of biofilm formation. Taken together, the technology and findings we describe here provide novel, quantitative insights into bacterial heteroresistance, a key step toward understanding antibiotic resistance and developing new tools to prevent further escalation of the antibiotics crisis.

## Results and Discussion

### Analysis of heteroresistance using droplet microfluidics

To capture the growth patterns of isolated single bacterial exposed to antibiotics, we encapsulated bacteria into water-in-oil droplets. We used a model weakly beta-lactam-resistant organism, *Escherichia coli* DH5α, carrying a TEM-20 beta-lactamase gene on a plasmid. This strain also harbours a second plasmid with yellow fluorescent protein (YFP) for detection. To assay resistance, we measured the minimum inhibitory concentration (MIC) of an antibiotic that prevents bacterial proliferation^20^.

**Figure 1:**
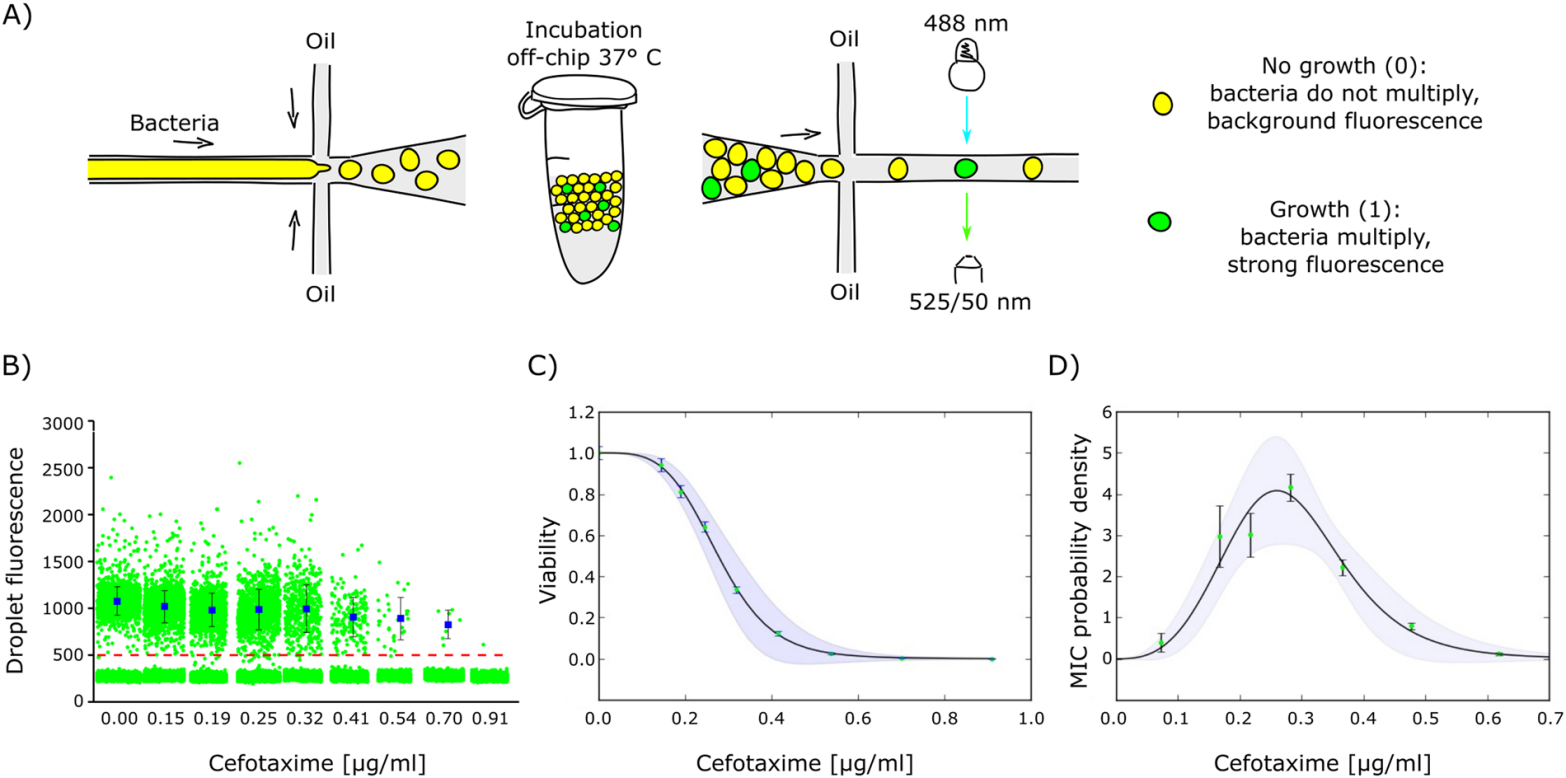
Cefotaxime reveals an *E. coli* heteroresistance pattern at the single-cell level. (**A**) Microfluidic workflow for the single-cell droplet assay in which an aqueous phase (consisting of bacteria, medium, and antibiotics) are encapsulated in surfactant-stabilized water-in-oil droplets. Each antibiotic concentration is screened in a separate library. During incubation, encapsulated bacteria start proliferating and synthesizing YFP, unless growth is inhibited by the antibiotic. After incubation, the fluorescence readout of each droplet is acquired with confocal microscopy. In principle, the assay is ‘digital’: the bacterium either grows (1-positive) or does not (0-negative). (**B**) Signal intensities of each droplet in the experiment (~10000 droplets per antibiotic concentration of which ~1500 droplets contained bacteria). Red dashed line at relative fluorescence value 500 marks the threshold for positive droplets. Blue rectangles show the average signal of positive droplets, with standard deviation as error bars. (**C**) Cell viability (fraction of positive droplets normalized by the value for the experiment without antibiotic, ${{f}}_{+}\frac{{c}}{{{f}}_{+}}(0)$) as a function of antibiotic concentration ***c***. Error bars are discussed in Fig. S5. Continuous line represents $1/[1+{({c}/{{c}}_{{s}})}^{{\boldsymbol{\alpha }}}+{{a}}_{1}{({c}/{{c}}_{{s}})}^{2{\alpha }}]$, with fitting parameters ${{c}}_{{s}}=(0.29\pm 0.029)\,{\mu }{g}/{ml}$, ${\alpha }=3.78\pm 0.76$, and ${{a}}_{1}=0.26\pm 0.52$ determined by the least-square method. Errors and the error propagation formula applied to the fit determine the shaded area. (**D**) Probability distribution of individual MICs in the population obtained from a numerical derivative of the data points in (**C**). Continuous line represents the negative derivative of the fit from (**C**) (the probability distribution of single-cell MICs in the population). The shaded area shows errors obtained from the error propagation formula applied to the negative derivative of fit from (**C**).

We used microfluidic chips with flow-focusing geometry to generate libraries of monodisperse 2-nl droplets that act as separate, miniature test tubes (Fig. 1A). We encapsulated cells with the beta-lactam antibiotic cefotaxime (formulating a separate droplet library for each antibiotic concentration), incubated the bacteria overnight, and screened droplets for increased fluorescence caused by fully grown (saturated) colonies (see Figs. S1–S4 for more detailed information about droplet generation and analysis). The term ‘colony’ here captures an accumulation of microbes in a droplet, usually occurring as a clone of a single original organism^16,21^. Therefore, a ‘single cell’ is equivalent to a single colony-forming unit (CFU). One bacterium per 2-nl droplet is equivalent to 5 × 10^5^ CFU/ml, which is the standard for MIC tests^20,22^.

Antibiotic susceptibility screening in droplets constitutes a digital assay^23,24^ in which the outcome of each discrete experiment in droplet is binary: ‘1-positive’ with detectable bacterial growth, or ‘0-negative’ without growth. We split the starting cell suspension (~1 × 10^5^ CFU/ml) into droplets. Of the total number of droplets, *N*, some are seeded with bacteria. Similar to a standard plating experiment, while we cannot visualize individual cells, we can count the number of ‘positive droplets’, *N*_+_, after incubation. We use the fraction of positive droplets *N*_+_/*N* in experiments without antibiotics to calculate the rate of bacterial encapsulation inside droplets. In digital assays^24^, this process is described by the Poisson distribution with an average encapsulation rate *λ* = −ln(1 − *N*_+_/*N*).

In order to measure the distribution of antibiotic susceptibility of single cells, we assume that the vast majority of non-empty droplets contain only a single cell. To minimize the probability of encapsulating two or more cells in a single droplet, the starting inoculum suspension must be diluted so that the encapsulation rate λ (average number of bacteria per droplet) is 0.1–0.3 or lower^24^. In our case, λ was ~0.18, and for each antibiotic concentration we screened libraries containing ~1,500 non-empty droplets.

For each library with a given antibiotic concentration, *c*, we count the total number of droplets, *N*(*c*), and measure number of positive droplets, *N*_+_(*c*). The fraction of positive droplets is denoted by *f*_+_(*c*) = *N*_+_(*c*)/*N*(*c*); see Fig. S5 for error analysis. Further, in order to analyse the response of bacteria to antibiotic, we normalize the number of positive droplets by their number in the absence of antibiotic, to obtain the fraction of individual cells that proliferate as a function of antibiotic concentration: *F*(*c*) = *f*_+_(*c*)/*f*_+_(0).

We observed that our model isogenic bacterial population exhibited a high degree of phenotypic growth variability between individual cells. Growth inhibition decreased gradually as the antibiotic concentration increased, with no sharp (Heaviside step function) transition between ‘growth’ and ‘no growth’ (Fig. 1B,C). Partial growth inhibition was evident at antibiotic concentrations as low as 0.15 µg/ml, but the lowest concentration yielding maximum inhibition was more than 10-fold higher, at 2 µg/ml (Fig. 1C). At the same time, colony density (indicated by fluorescence intensity in positive droplets) remained steady even when the fraction of positive droplets substantially dropped (Fig. 1B). This observation suggests that bacterial proliferation in droplets is a binary stochastic variable: individual cells either grow into colonies or they do not. Thus, we can reliably translate the fraction of proliferating cells in a droplet into the probability of single cells proliferating in a population.

We propose to employ the probability distribution density, *p*(*MIC*), of cells exhibiting a given MIC as a well-defined measure of heterogeneity. This probability can be calculated directly from the identity $F(c)={\int }_{c}^{\infty }\,p(m)dm;$ bacteria that proliferate at a given antibiotic concentration *c* are characterized by MIC level *m* ≥ *c*. Thus, $p(c)=-\frac{d}{dc}F(c)$ (Fig. 1D).

The resistance profile described here is heteroresistance by definition: the lowest antibiotic concentration inhibiting 100% of bacterial proliferation is >8-fold higher than the highest non-inhibitory concentration^13^, similar to the heteroresistance pattern on Fig. 1C. The term heteroresistance is used very liberally and often confusingly in the literature^13^, with synonyms like ‘transient resistance’^25^ and ‘dose-dependent persistence’^11^. This confusion is why we prefer the term ‘clonal heteroresistance’, introduced recently by Band *et al*.^15^, to emphasize our description of resistance profiles in isogenic bacterial populations originating from a single colony.

It is highly unlikely that the emergence of spontaneously resistant mutants explains the variety in resistance detected here, because the mutation rate in bacteria is orders of magnitude lower^26^ than the fraction of growing bacteria in this experiment. Nor can this diverse resistance profile be explained by the presence of time-dependent persister cells, as in our setup the bacteria grow under constant antibiotic exposure^11^.

To the best of our knowledge, this is (1) the first quantitative description of the diversity of clonal heteroresistance at the single-cell level, and (2) the first portrayal of the resistance probability distribution of single bacterial cells in an isogenic population. Recently, Lyu *et al*.^19^ used a similar droplet-based microfluidic setup to describe an increase in resistance at the single-cell level. However, they did not analyse the resistance profile of an isogenic bacterial population; rather, they evaluated the emergence of sub-populations with higher resistance during antibiotic exposure, which can be caused by the acquisition of novel genetic mutations^19^. Previously, Eun *et al*. unknowingly captured a heteroresistance pattern with rifampicin in a gel-droplet assay, but they did not draw attention to it^27^. When we reanalysed data from Fig. 3 of Eun *et al*.^27^, we noted that their probability distribution was similar to ours (Fig. S7).

We speculate that the clonal heteroresistance detected here most likely arises from differences in the expression of TEM-20, an extended-spectrum beta-lactamase, or more globally from differences in the entire transcriptome of resistant cells. In our model system, expression of the gene encoding TEM-20 is likely noisy^28^, as it is modulated by at least two stochastic components: the copy number of the plasmid carrying TEM-20 and the general variability of gene expression^10,29^. Recently it was reported that a copy-number increase in resistance genes most often underlies heteroresistance which can lead to non-optimal antibiotic treatment regimens and relapse of infections^14^. Heterogeneity in the expression of drug-resistance proteins like beta-lactamases^30^ or multi-drug efflux pumps^31^ have also been strongly linked to heteroresistance. As we discuss below, our method enables estimation of the degradation rate of antibiotics, with results consistent with the above assertions.

### Modulation of antibiotic susceptibility with increasing inoculum density

In a complementary experiment, we investigated how the susceptibility of *E. coli* to an antibiotic changes with increasing inoculum density. Beta-lactam antibiotics are often subject to the inoculum effect, in which the efficiency of a drug depends on the starting inoculum density of the bacteria^32^. The inoculum effect is often overlooked in traditional MIC assays due to inaccuracies in setting the inoculum density to recommended levels via conventional measurements of optical density^22^. Our digital MIC assay overcame this hurdle.

**Figure 2:**
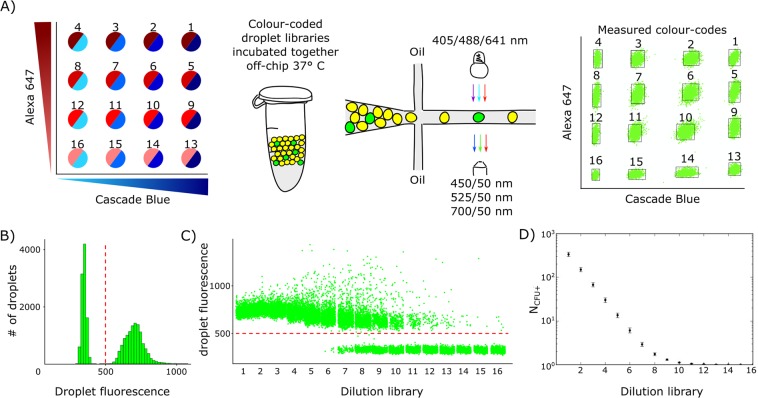
A colour-coded droplet virtual array reveals inoculum density. (**A**) Schematic for colour-coding bacterial densities. Cascade Blue and Alexa 647 dyes are represented in the virtual array as a 4 × 4 concentration matrix of 16 colour-code combinations (darker colour corresponds to higher dye concentration). Two-fold serial dilutions of bacteria are colour-coded and introduced sequentially into the microfluidic system for droplet generation. Colour-coded droplet libraries are pooled into a single master library. After incubation, droplet fluorescence is acquired in three separate channels (three arrows). Droplets are gated to 16 bins in a virtual array based on their Cascade Blue and Alexa 647 signal intensities (virtual array with ~22000 droplets). (**B**) Histogram of the pooled droplet signals, with bacterial growth in the green channel. Red dashed line denotes the threshold between negative and positive droplets. (**C**) Plot of bacterial growth (relative fluorescence of droplet in green channel) measured separately in each droplet. Droplets are sorted according to their colour-code allocation in the virtual array (the same data as in (**A**,**B**). Note the substantial population of droplets with high fluorescence intensity (near 1000 and above). This phenomenon is explained in “clumping” section. (**D**) Average number of bacteria in non-empty droplets (*N*_*CFU*+_) in various virtual array libraries (Fig. S5).

To precisely estimate bacterial densities and to measure inoculum effect, we harnessed the ‘virtual array’ strategy described by Abate *et al*.^33^ to pool droplet libraries for easier downstream handling and analysis. We prepared series of 16 two-fold dilutions of our YFP-carrying *E. coli* and labelled each dilution with two fluorescent dyes: Cascade Blue and Alexa 647 (both from Thermo Fisher Scientific; Fig. 2A). Immediately after generation, the 16 colour-coded libraries were pooled into a single 1.5-ml test tube for overnight incubation at 37 °C.

We calculated the initial inoculum density in the virtual array using a digital counting algorithm and equations described previously^23,34^. We set a fluorescence threshold to distinguish between growth-positive and growth-negative droplets (Fig. 2B). Next, we separately investigated each library in a virtual array to calculate the corresponding fractions of positive droplets (Fig. 2C). We calculated the average droplet occupation event for each library as ‘CFU/positive bacteria-containing droplet’ (*N*_*CFU*+_) (Fig. 2D). See Fig. S5 for more detailed explanation of the calculations. The average *N*_*CFU*+_ spanned over two orders of magnitude, from 1 to ~338 per droplet (Fig. 2D), which translates to an inoculum range between 5 × 10^5^ and ~1.7 × 10^8^ CFU/ml conventionally.

**Figure 3:**
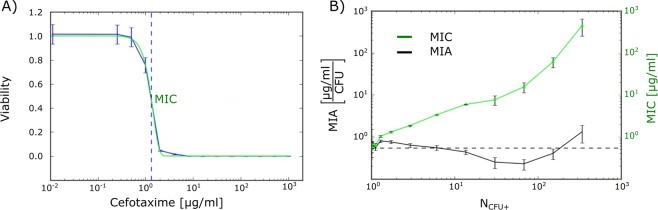
The inhibiting amount of cefotaxime per bacterium remains stable over a wide range of bacterial densities. (**A**) Calculation of MIC using a Gompertz function fit (green line) with bacterial density ${{N}}_{{CFU}+}=1.73$. Blue vertical dashed line shows the position of the MIC where the Gompertz fit crosses the 0.5 viability fraction in droplets. (**B**) Comparison of MIC (green) and minimum inhibitory amount (MIA; black) for various inoculum densities. MIA is defined as the amount of antibiotic per bacterium inside non-empty droplets normalized by the droplet volume: ${MIA}={MIC}/{{N}}_{{CFU}+}$. Dashed line shows the average MIA.

To investigate the inoculum effect, we produced a series of separate virtual arrays with cefotaxime concentrations ranging from 0.25 to 1024 μg/ml, with a twofold dilution between samples. We used one virtual array without antibiotics as a control. For each bacterial density, we fit the positive droplet fraction data with the Gompertz function^35,36^. Then, we calculated the MIC as the antibiotic concentration where the Gompertz fit crosses the 0.5 viability fraction in droplets (Fig. 3A), meaning that the inhibition of bacteria growth occurs in 50% of the droplets.

We observed a strong inoculum effect^22^: the MIC rose more than 8-fold as the bacterial density increased by two orders of magnitude (Fig. 3B). This was expected, as similar findings have been reported previously with the same bacterial strain^32^. We also detected a similar inoculum effect in a coinciding experiment with a different microfluidic setup using smaller droplets and different growth medium^37^. Interestingly, when we examined growth inhibition *per capita*, $MIC/{N}_{CFU+}$ in our experiment, the amount of cefotaxime needed to suppress growth remained stable around 0.5 µg/ml/CFU (Fig. 3B).

Together, these data suggest that the amount of antibiotic per bacterium is the crucial parameter that determines growth inhibition: MIA = $MIC/{N}_{CFU+}$, where MIA is the minimum inhibitory amount of antibiotic normalized by the droplet volume. We suggest including this parameter in future assays of antibiotic susceptibility. With limited beneficial interactions between bacteria during antibiotic exposure, the MIA remains stable regardless of bacterial inoculum density. However, if MIA were to significantly increase during incubation, then a synergistic effect may be taking place as bacteria fight the antibiotics.

We used the MIA to estimate the maximal rate of degradation of cefotaxime by beta-lactamases in our bacteria. We assumed that MIA corresponds to the highest concentration of antibiotic that can be hydrolysed by enzymes within a time interval, ${t}_{d}\approx 20\,min$, which approximates (to an order of magnitude) the division time of this species under normal conditions. Therefore, the rate of degradation of the antibiotic by a single bacterium inside a droplet of volume $v=2\,nl$ is ${V}_{max}=MIA\times V/t=8.3\times {10}^{-19}\,kg/s$. A cefotaxime molar mass of 455 g/mol yields 10^6^ cefotaxime molecules degraded per second by a single bacterium. This crude estimate is of similar order as *V*_*max*_ obtained for the same strain by Artemova *et al*.^32^, who previously used Michaelis-Menten kinetics to calculate enzyme degradation.

An additional advantage of encapsulating bacteria in droplets is that the assay becomes insensitive to fluctuations in inoculum density, in our case below an encapsulation rate λ of ~0.25 (Fig. 2D). Under these conditions, the inoculum density used to prepare the droplets does not need to be controlled precisely, as the effective maximum density of bacteria in droplets will always be 1 bacterium per droplet. Our 2-nl setup therefore ‘locks’ the assay into the standard 5 × 10^5^ CFU/ml inoculum density recommended by CLSI and by EUCAST, opening new ways to design novel assays of ‘inoculum density-resistant’ antibiotic susceptibility.

### Clumping of bacteria at sub-inhibitory antibiotic conditions

Unexpectedly, we found that our droplet-based system is also an excellent tool for high-throughput analysis of bacterial clumping. We first noticed clumping in the control virtual array, where we observed outlier droplets presenting fluorescence above the usual intensities for positive droplets (Fig. 2C). Closer study of relevant confocal images (Fig. S8) revealed clusters of high-intensity pixels inside those droplets, which we interpreted as bacterial clumps. We measured the relative size of the clumps by dividing the total area of clumps within a droplet by the droplet area in confocal cross-section image (Fig. S8).

Clumping was intensive in a certain fraction of droplets above the 90th percentile in terms of the clump-to-droplet area ratio; we used this value (clump covering ~1% of the droplet in the image) as a threshold for intensive clumping (Fig. S8). In ~ 80% of clumping cases, we observed a single dominant clump inside the droplet. Although the droplet assay and confocal imaging were not optimized for clumping analysis and some clumping events may have been overlooked, any potential mis-representation would be systematic and does not affect our overall findings or their trends (Fig. S2).

**Figure 4:**
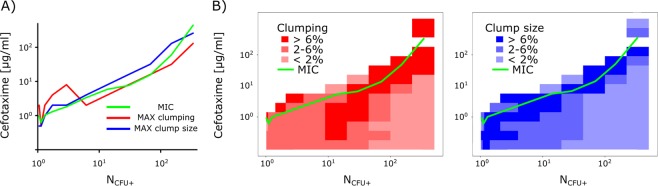
Cefotaxime modulates the clumping of bacteria. (**A**) At various inoculum densities, both the highest clumping (red) and the greatest size of clumps (blue) occur near sub-inhibitory cefotaxime conditions (green). (**B**) Heat maps of relative clumping rates (red) and relative clump sizes (blue)) in the matrix of different bacteria densities (X-axis) and cefotaxime concentrations (Y-axis). Green line shows the approximate MIC in these experiments (same data as on Fig. 3B).

Reanalysis of the inoculum density data revealed that bacterial clumping is modulated by cefotaxime and is highest at sub-inhibitory drug conditions near the MIC (Fig. 4A). Clumping is also modulated by the inoculum density: at low antibiotic concentration, droplets harbouring a low density of bacteria exhibit more clumping than droplets with high bacterial density (Fig. 4B, bottom). Full datasets for clumping events and clump sizes appear in Table S3 and Table S4, respectively. Recently, we observed similar clumping trends in smaller droplets using a different growth medium^37^.

An increased tendency for bacteria to clump or agglomerate under sub-MIC conditions was previously described for beta-lactams and aminoglycosides during early biofilm formation in several species^38,39^. We hypothesize that our droplet assay captured the early stages of biofilm formation that are modulated by cefotaxime. Since we used a system in which water droplets were stabilized in oil using surfactant, there were no solid surfaces or crevices where bacteria/clumps could attach to form a biofilm, leaving them in a pre-biofilm clumping stage. Future investigations could add extra beads^40^ or surfaces^41^ into droplets to encourage bacterial attachment. With capabilities beyond those of traditional microscopy and well-plate systems, our high-throughput experimental design could be advantageous for further basic research into clumping, as well as research into the initiation and progression of biofilm formation and how it impacts antibiotic resistance^42^. This concept could be extended to test various antimicrobial coatings and their properties.

## Conclusion

The droplet-based digital MIC screen described here enabled us to carry out the first quantification of clonal heteroresistance in an isogenic bacterial population and to determine the probability distribution of MICs. Such robust measurement of heterogeneity is crucial for understanding how sub-populations of bacteria survive when exposed to antibiotics and potentially accumulate resistance-increasing mutations. Importantly, we observed that the relative amount of antibiotic needed to stop bacteria growth was quite stable over a wide range of inoculum densities; near these inhibiting conditions, bacteria increasingly clumped together. Our microfluidic solution is also promising for high-throughput investigations of early biofilm formation and its prevention, which is crucial for combating antibiotic resistance. We predict that the droplet-based technology developed here will soon be relevant and directly usable in clinical contexts, as there have been rapid improvements in applying label-free detection principles for droplet-based analysis^16^.

## Methods

### Bacteria

For these experiments, we used an *Escherichia coli* DH5α strain harbouring two plasmids^32^ (a kind gift from Professor Jeff Gore, MIT, USA). The first plasmid carries a constitutively expressed YFP gene; we used 50 µg/ml of piperacillin sodium salt (Sigma-Aldrich, Germany) for selection. The second plasmid carries the gene encoding TEM-20, with 50 µg/ml of kanamycin sulfate (Sigma-Aldrich) used for selection. All experiments, both in bulk and in droplets, were carried out in LB Lennox medium (Roth, Germany). Antibiotic susceptibility experiments were done with cefotaxime sodium salt (Sigma-Aldrich). Overnight cultures were diluted to the required densities with fresh medium before droplet experiments were begun, and cells were kept at 4 °C until they were encapsulated in droplets.

### Microfludics

Fabrication of the microfluidic chips used in this work has been described elsewhere^23,43^. We employed separate chips for droplet generation and for fluorescence analysis (Fig. S1). We used Novec HFE-7500 fluorocarbon oil (3M, USA) with 2% PFPE-PEG-PFPE surfactant (synthesized in accordance with^44^). To control the flow of oil and reagents in the microfluidic devices, we used a rotAXYS positioning system and neMESYS syringe pumps (both from Cetoni, Germany). Droplets were generated at ~800 Hz and were analysed at ~400 Hz. Conventional 1.5-ml test tubes were used for off-chip incubation at 37 °C. Labelled dextran conjugates were employed in our virtual array setup to label bacteria densities (Cascade Blue^TM^ and Alexa Fluor 647^TM^, Thermo Fisher Scientific, USA).

### Fluorescence measurements and data analysis

We measured the fluorescence of droplets in our droplet-reading chip that was mounted on the stage of an A1R confocal microscope (Nikon, Japan). The excitation and detection settings were as follows: Cascade Blue, 405 nm and 450/50 nm; YFP, 488 nm and 525/50 nm; Alexa 647, 641 nm and 700/50 nm. Note: because of the hardware setup in the confocal, we used GFP settings to obtain fluorescence of YFP. Raw images were analysed with Nikon NIS-Elements AR 3.2 software and results were exported as.txt files. Further analysis was carried out with MS Office Excel (Microsoft, USA) with the Real Statistics Resource Pack (http://www.real-statistics.com/) or with custom LabVIEW (National Instruments, USA) scripts. Droplet signals reflect the peak relative fluorescence intensities allocated to each droplet (Figs. S2, S3).

### Virtual arrays

Each virtual array corresponds to a series of 16 two-fold dilutions of the *E. coli* inoculum. The first dilution was prepared by refreshing the overnight bacterial culture with fresh medium at a 1:10 ratio. Sample loading and droplet generation were described previously^23^. In brief, we aspirated 3 ul of each colour-coded bacterial dilution into microfluidic tubing, spaced with an equal volume of an oil plug. Next, we compartmentalized the plugs into ∼2-nL droplets (the size distribution of droplets appears in Table S1) and pooled them as a ‘virtual array’ in a standard 1.5-ml test tube for incubation at 37 °C. After incubation, we analysed the droplet signals of the virtual array in three distinct fluorescence-detection channels.

We positioned the droplets in the virtual-array matrix based on their Cascade Blue and Alexa 647 signal intensities. Then we identified each cluster using custom-made Labview script, determined the bounding box encapsulating the points within each cluster, and assigned a colour-code number to each gated droplet cluster. The colour-coding histogram in Fig. 2A contains data from ~22,000 droplets (~1,400 per colour-coded bacteria dilution). In our virtual-array experiments we repetitively identified colour codes for >97% of the droplets (Fig. S3).

### MIC and MIA calculations

We fit the data for each bacterial density with the Gompertz function^35,36^, which is a two-parameter function:

$$\phi =\exp (-{(\frac{c}{{p}_{1}})}^{{p}_{2}}),$$

where *c* is the concentration of antibiotic (argument of the Gompertz function), *p*_1_ is concentration at which the highest drop of *ϕ* is observed, and *p*_2_ determines the slope of the Gompertz function at *c* = *p*_1_. The parameters and their errors are determined by the least-square method. We define MIC as the concentration for which *ϕ* = 1/2, that is, ${c}_{MIC}={p}_{1}{(\mathrm{ln}2)}^{1/{p}_{2}}$. We estimate the error of *c*_*MIC*_ by the minimum error obtained by the error propagation formula applied for ${c}_{MIC}={p}_{1}{(\mathrm{ln}2)}^{1/{p}_{2}}$ or by the difference between concentrations of the antibiotic closest to *c*_*MIC*_. MIA is determined by $MIA\equiv v{c}_{MIC}/{N}_{CFU+}$, where *v* is the droplet volume. Error for MIA is determined by the error propagation formula for the above equation. The probability distribution of individual MICs is related to the normalized fraction of positive droplets, ${\int }_{c}^{\infty }\,p(m)dm={f}_{+}(c)/{f}_{+}(0)$. Taking the derivative of this formula yields $p(c)=-\,\frac{d}{dc}{f}_{+}(c)/{f}_{+}(0)$. To calculate the derivative, we use the data points from Fig. 1C for concentrations *c*_*i*_ according to $p(({c}_{i+1}+{c}_{i})/2)=(\frac{{f}_{+}({c}_{i+1}\,)}{{f}_{+}(0)}-\frac{{f}_{+}({c}_{i})}{{f}_{+}(0)})/({c}_{i}-{c}_{i+1})$. Errors of the above probability distribution are determined with the error propagation formula from the errors of ${f}_{+}({c}_{i}\,)$, ${f}_{+}({c}_{i+1}\,)$ and ${f}_{+}(0\,)$.

## Supplementary information

Supplementary Information.
