# Supplementary material for: Droplet-based digital antibiotic susceptibility screen reveals single-cell clonal heteroresistance in an isogenic bacterial population
Source: Sci Rep. 2020 Feb 24;10:3282. doi: 10.1038/s41598-020-60381-z (PMC7039976; doi:10.1038/s41598-020-60381-z)
Supplement: Supplementary file 1 — Supplementary Information. [file 41598_2020_60381_MOESM1_ESM.docx]

Supplementary Information for

**Droplet-based digital antibiotic susceptibility screen reveals single-cell clonal heteroresistance in an isogenic bacterial population**

Ott Scheler, Karol Makuch, Pawel R. Debski, Michal Horka, Artur Ruszczak, Natalia Pacocha, Krzysztof Sozański, Olli-Pekka Smolander, Witold Postek and Piotr Garstecki

Corresponding authors: Ott Scheler and Piotr Garstecki

Email: *ott.scheler@taltech.ee, garst@ichf.edu.pl*


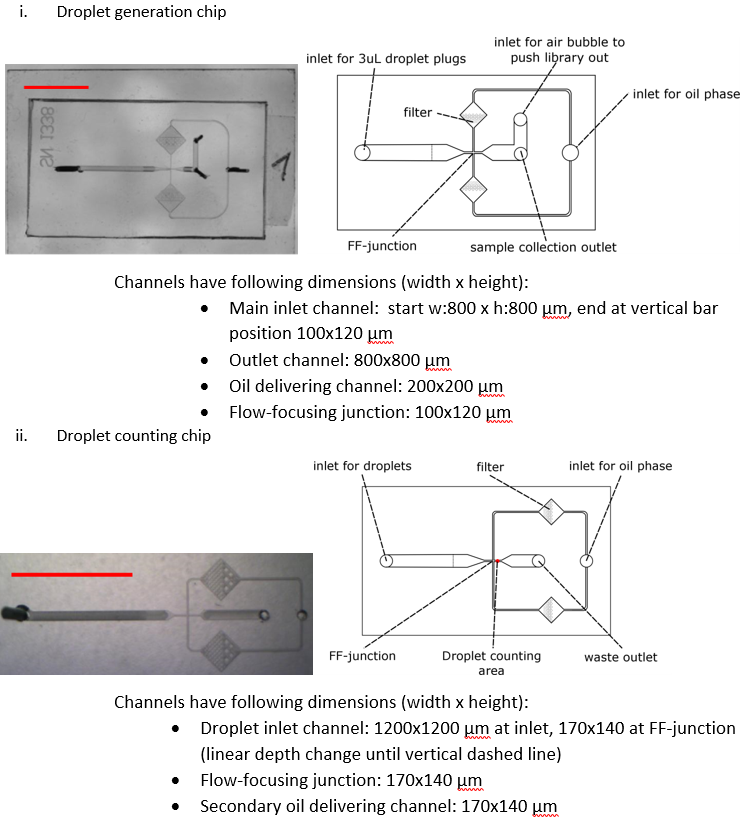


**Fig. S 1.** Microfluidic chips. Schematics of i) droplet generation and ii) droplet counting chip. Red scale bar in the figures is 1 cm. Schematics on the right are representative and do not show the exact size or proportions of the geometries. The exact CAD files are available upon request from the authors.

**Table S 1**. Droplet size and variation. For measuring the size dispersion of the droplets that were generated from 3µL plugs, we recorded the droplet formation using Photron Fast-Cam 1000K (Japan) camera that was mounted on a Nikon SMZ1000 stereoscope (Japan). We measured the whole droplet formation process of a 3 µL plug. We picked randomly four different plugs from a dilution series. We measured the area of each generated droplet in Image J software and used this data to calculate the volume of each droplet according to the model described previously.(1–3) Briefly, the volume can be calculated by the following formula: V=(π/12)[2D3-(D-h)2(2D+h)], where D is the diameter of a droplet and h is the height of the channel.


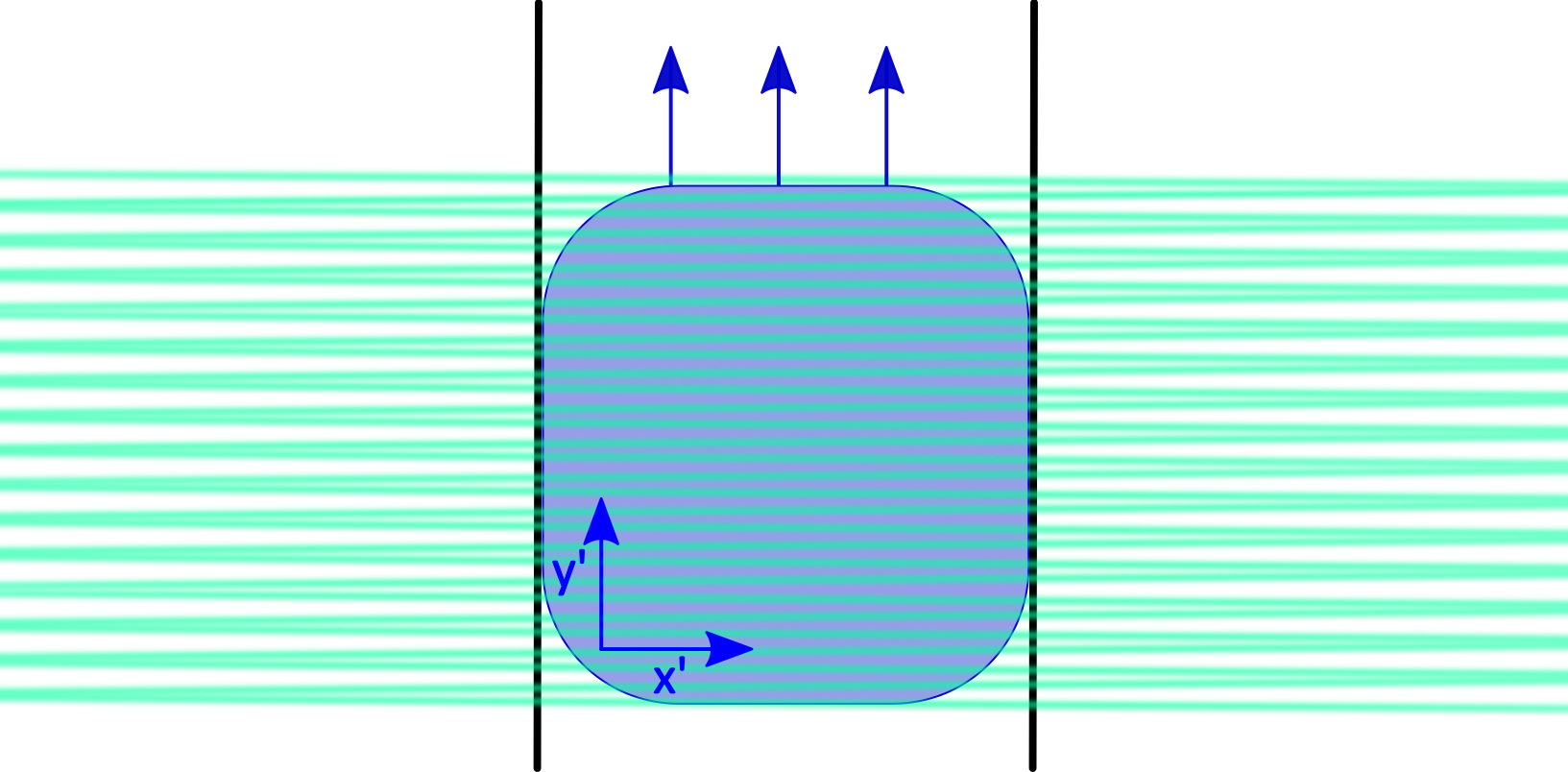


**Fig. S 2.** Confocal microscopy data acquisition. All confocal microscopy experiments were performed using a Nikon A1R setup, based on an inverted confocal microscope Nikon Eclipse Ti-E, equipped with the LU4A laser unit and four-channel detector unit (all by Nikon, Japan). A Nikon Plan Fluor 10x, NA=0.3 objective was used. The microscope was controlled via Nikon NIS-Elements AR 4.13 (64-bit) software. To maximize the throughput of the system, we acquired fluorescence data using a resonant scanner in a bi-directional single-line mode, with scanning path perpendicular to the microfluidic channel. This allowed for a recording speed of 15360 lines per second (pixel dwell of 0.1 μs, line time of around 65 μs). At 488 nm, the width of the beam at the focus plane (calculated as doubled 1/e2 radius) was around 1.4 μm. Taking the length of an average droplet in the channel of 145 μm and an average of 40 line scans across each droplet during its passage through the detection zone, we obtain a 37% coverage of the total cross-sectional area of a droplet with the scanning beam (see figure below). With a widely open pinhole (115 μm), excitation wavelength of 488 nm and a numerical aperture of the objective of 0.3, the thickness of the optical section (i.e. the axial dimension of the detection area) was 57 μm, as reported by the NIS software. This is 41% of the total height of the microfluidic channel. Thus, we can estimate that around 15% of the total volume of the droplet was effectively sampled during the fluorescence data acquisition. Importantly, the scanned lines were distributed evenly along the length of the droplets and the focus plane was set in the center of the channel height. Therefore, we can assume that the data we recorded for each droplet was representative for its whole volume. During data acquisition in line mode, the laser beam is only scanned perpendicularly to the channel (along the x axis). However, the droplet is constantly moving in the y direction. Therefore, in a droplet-bound frame of reference (x’,y’), this situation is equivalent to the laser beam scanning through consecutive sections of the droplet in a zig-zagging manner – as schematically depicted in the figure.


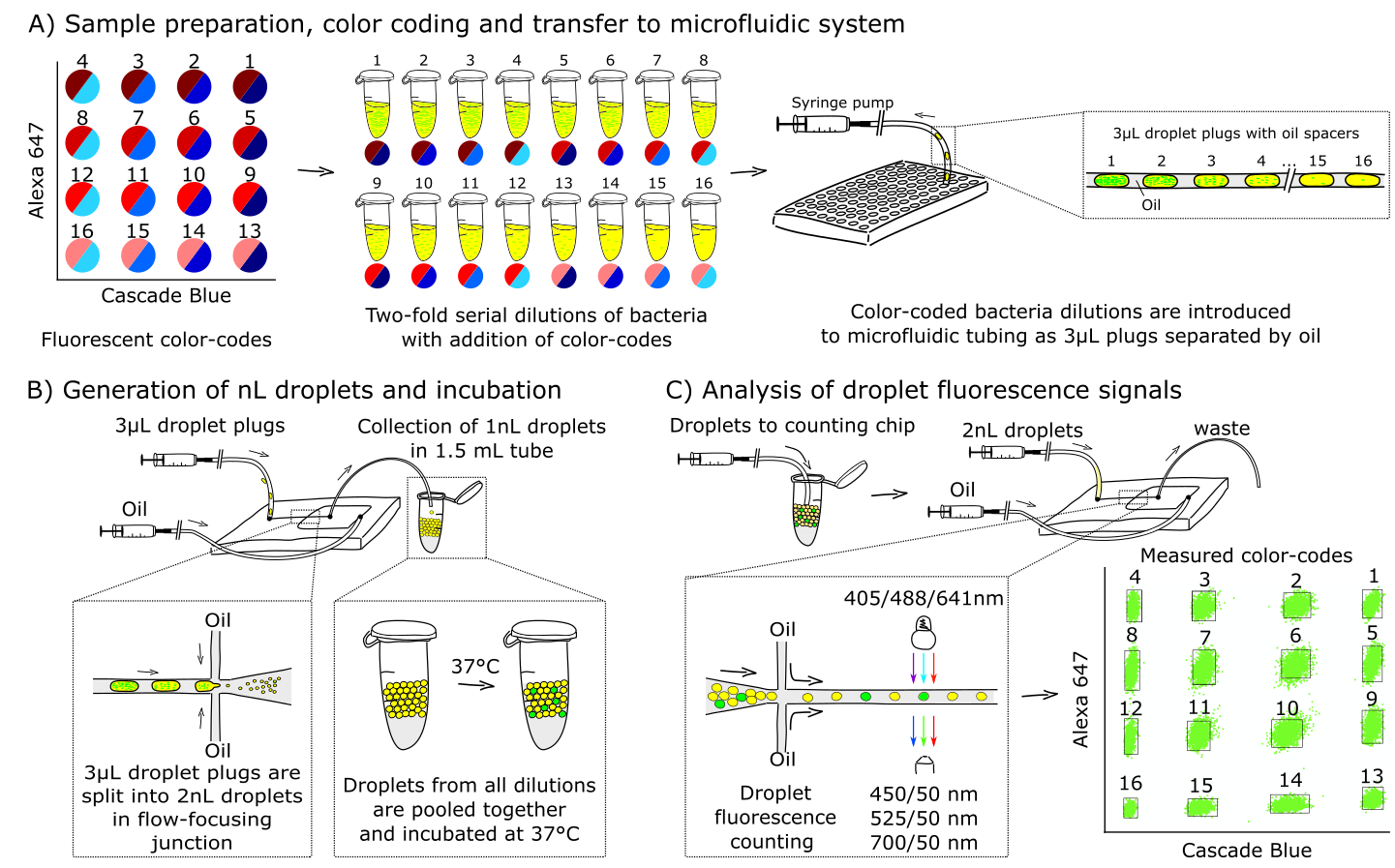


**Fig. S 3.** Color-codes were generated with Alexa 647 10000 MW and Cascade Blue 3000 MW dextran conjugate dyes (both from Thermo Fisher). The respective 4x4 dye matrix consisted of following concentrations: i) Cascade Blue (2, 15, 30 and 60 µg/mL) and Alexa 647 (0.5, 1.5, 3 and 6 µg/mL). Two-fold serial dilutions of bacteria were color-coded and introduced to microfluidic tubing as oil-separated 3 µl plugs. Plugs were split into 2 nL droplets and pooled together into a single library. After incubation the virtual array was inserted into the counting chip that was fixed in a confocal microscope stage to measure the intensity of fluorescence emitted by the droplets. For spacing the droplets during the measurement, an additional stream of carrier oil was introduced into the flow-focusing junction immediately before the acquisition area. Each droplet was then assigned “the address” in the virtual array based on its gating into the bounding box. First, raw image stack time-lapse .nd2 files were analyzed with Nikon NIS software and the results were exported as .txt files. Next, the files were analyzed with custom-made LabVIEW script to assign each droplet i) color-code address (Cascade Blue and Alexa Fluor 647 channels) and ii) bacteria fluorescence intensity (YFP channel). The LabVIEW script is available from the authors upon request. In brief, the script plots color-codes in the 2D space after which the droplets are assigned addresses according to their clustering into groups.


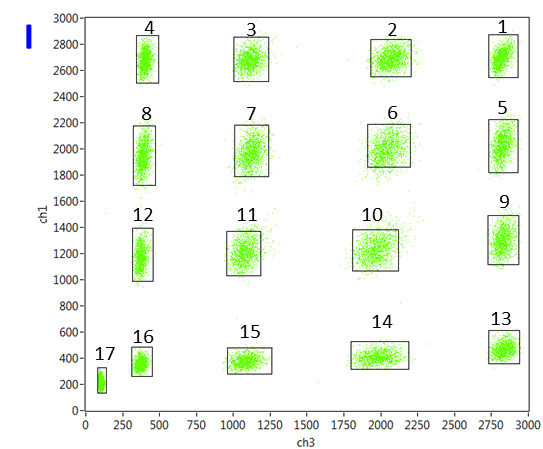

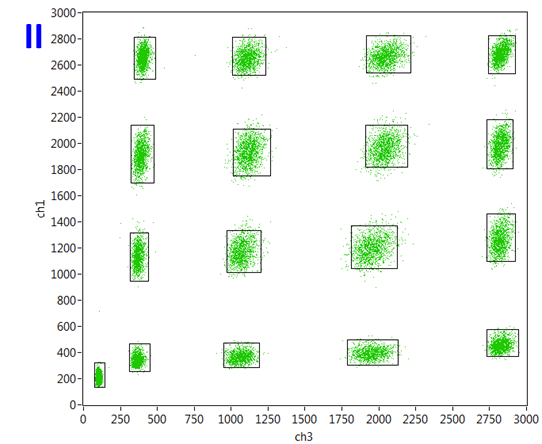


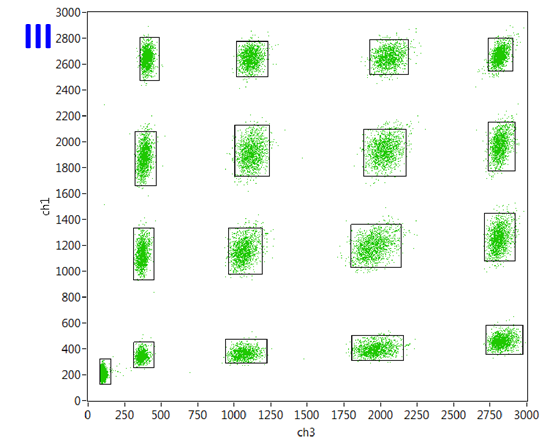

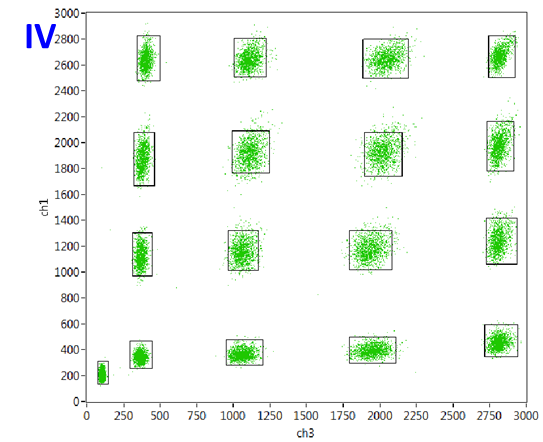


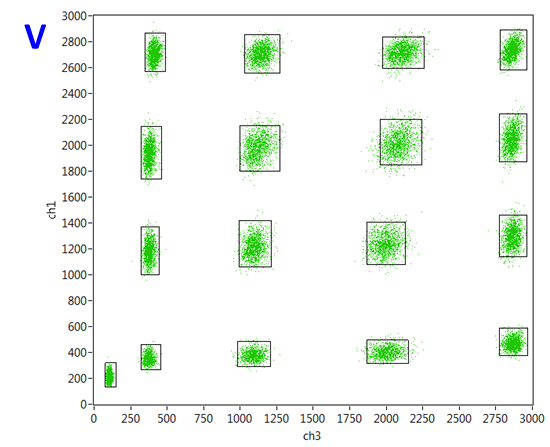

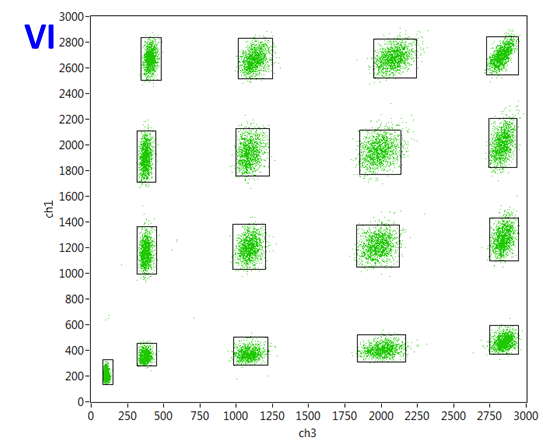


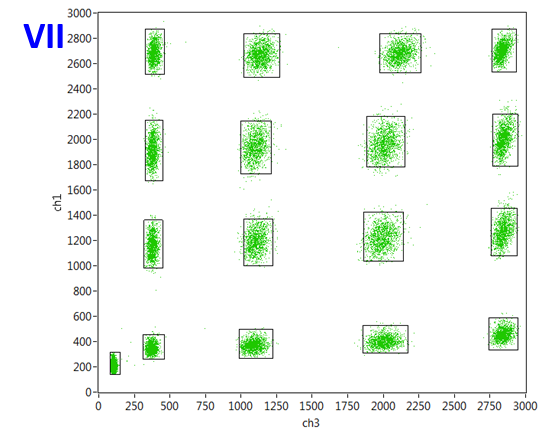

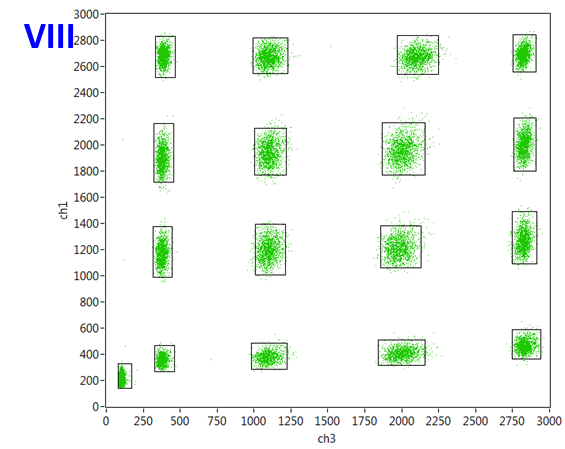


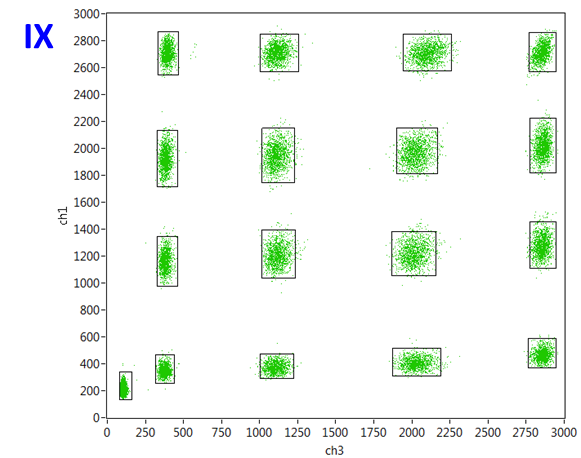

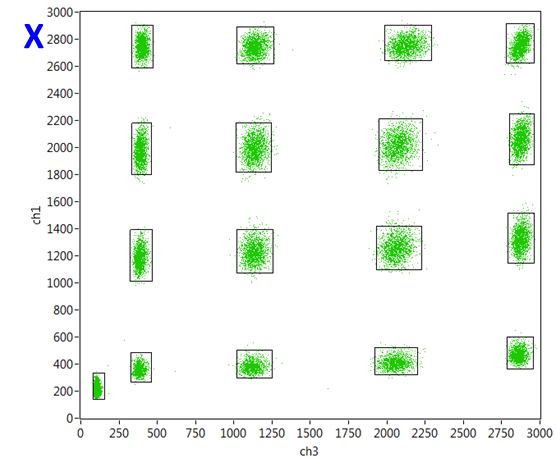


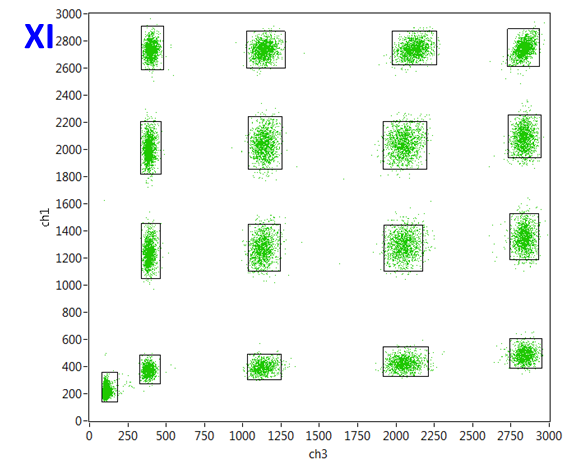

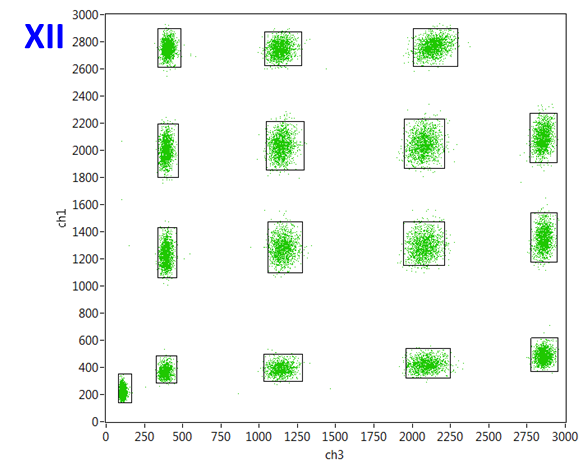


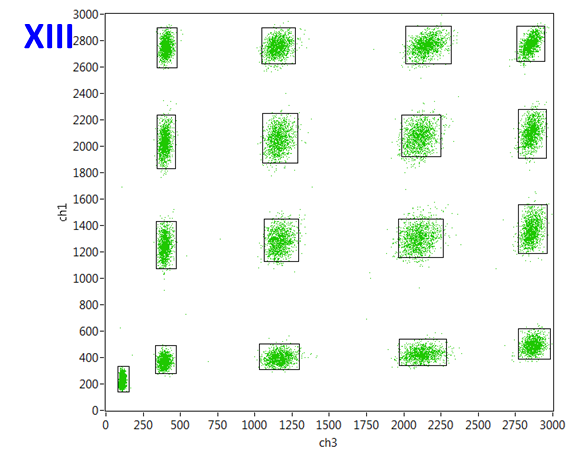

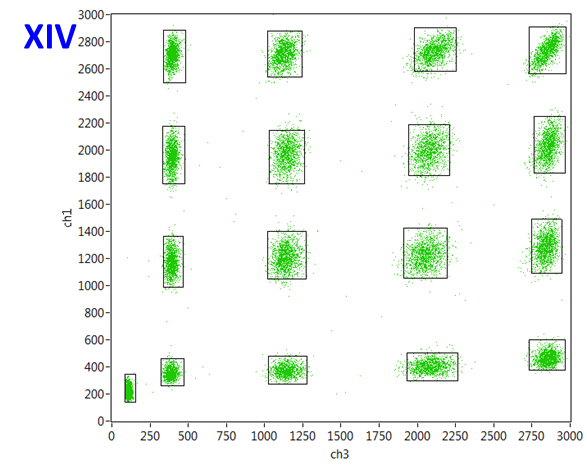


**Fig. S 4.** Plots showing the virtual arrays according to Cascade Blue (Ch1) and Alexa Fluor 647 (Ch2) intensities. Rectangular boxes mark the gating conditions for each color-code calculated by LabVIEW script. Color-codes from 1 to 16 represent bacteria densities from highest to lowest, respectively. Only droplets that fall within gated boundaries are used for further data analysis. Non-color-coded plugs with pure medium were added to the beginning and the end of plug train during droplet generation to minimize possible flow rate fluctuations. These droplets locate as color-code 17 in the bottom left and they are omitted from further analysis. Roman numerals I to XIV stand for different virtual arrays with increasing cefotaxime concentration from 0 to 1024 μg/mL, respectively. We note that color-code 1 is missing from virtual array XII due to the human error during droplet generation. However, this does not affect the scientific conclusions drawn in the manuscript.

**Table S 2.** Table showing the number of droplets analyzed per each virtual array with average number of droplets/color code shown. Last column shows the percentage of droplets that was gated and assigned with color-code address during the analysis.


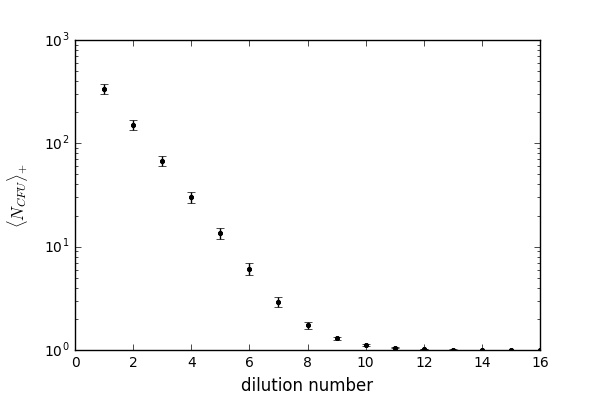


**Fig. S 5.** Number of bacteria inside droplets. We scan the droplets after incubation to measure how many droplets we have, $N,$ and the number of droplets which give a positive signal, $N_{+}$. The fraction of positive droplets is denoted by, $f_{+}\equiv N_{+}/N$. In further analysis we assume that in each droplet there is a random number of bacteria after the initial emulsification. Therefore, there is the probability $f_{+}$ that a droplet gives a positive signal (i.e. contains at least one bacterium after the emulsification) and the probability $1-f_{+}$ that a droplet gives a negative signal (contains no bacteria after the emulsification). From this perspective our experiment with droplets relies on performing $N$ Bernoulli trials with the probability of success equal to $f_{+}$. It follows from the central limit theorem that in the limit of large number of droplets (i.e. large number of Bernoulli trials) the fraction of positive droplets have a Gaussian distribution with the average $f_{+}$ and the dispersion $\sigma\_f\_+ =\surd(f_{+} (1-f_{+} )/N)$. The central limit theorem cannot be applied for the case when $f_{+}=0$ or $f_{+}=1$. In this case we estimate error of the measurement of $f_{+}$ using Bayesian inference by, $\sigma_{f_{+}}=1/N$. The fraction of positive droplets can be used to calculate the average number of bacteria inside a droplet, $N_{CFU}\equiv N_{bact}/N$, where $N_{bact}$ denotes total number of bacteria inside all droplets. It is possible after assumption that bacteria are closed in droplets according to Poisson statistics.(4–6) It follows that $N_{CFU}\left( f_{+} \right)=-\ln\left( 1-f_{+} \right)$. For the interpretation of our data we use the average number of bacteria inside droplets which gives a positive signal, $N_{CFU+}\equiv N_{bact}/(N-N_{+}). \_l iven by iven by signaledta we used nian statistics$ In terms of the fraction $f_{+}$ it is given by the following formula, $N_{CFU+}\left( f_{+} \right)=-[\ln\left( 1-f_{+} \right)]/f_{+}$. The above formulas are used to determine $N_{CFU}\left( f_{+} \right)$ and $N_{CFU+}\left( f_{+} \right)$ in our experiments shown in Fig. 1 from the main text. Their errors are determined from the error propagation formula(7) and the error of $f_{+}$, i.e. $\sigma_{f_{+}}$. The above two expressions for $N_{CFU}\left( f_{+} \right)$ and $N_{CFU+}\left( f_{+} \right)$ lead to the relation$N_{CFU+}=N_{CFU}/\left( 1-exp\left( -N_{CFU} \right) \right)$. The above formula for average number of bacteria in droplets cannot be used when all droplets give a positive signal. It happens in some of our 16 dilutions. To calculate density in all dilutions, we assessed first only the libraries that contained less than 100% positive droplets (libraries 6-16). For these libraries we determined $N_{CFU}$ values with errors using the method described in the previous paragraph. As the consecutive libraries were serially diluted, the estimated numbers of bacteria provide also the dilution ratio between the libraries, $N_{CFU,i}=n_{1}/x^{i-1}$. We calculate $n_{1}$ and $x$ parameters by fitting this curve to the numbers of bacteria (and their errors) obtained for the libraries 6-16 using least squares method. The estimated dilution ratio between the libraries was equal to $x=2.23\pm0.03$ and $n_{1}=340\pm40$ which we use to calculate densities in all samples. For each experiment we also determine $N_{CFU+}$ from $N_{CFU}$ (and its error from the error propagation formula), as described at the end of the previous paragraph.


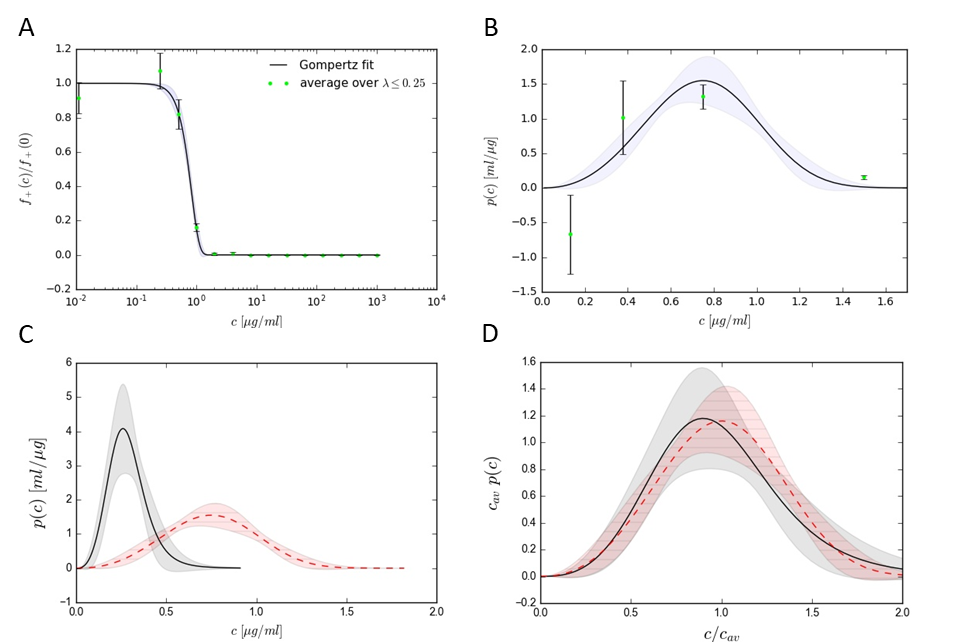


**Fig. S 6.** Single cell MIC in color-coding experiments. As described in the main text we perform experiments for sample with encapsulation rate λ≈0.18 shown in Fig. 1 and experiments for different λ with color coding technique described in Fig. 2 and 3. Because the latter experiments also contains 7 samples with small encapsulation ratios, λ≤0.25 which are appropriate for determination of single cell MICs, we use those experiments to determine probability distribution of individual MICs presented below. A) Viability fraction averaged over 7 experiments with encapsulation rates, $\lambda=0.002, 0.004, 0.01, 0.022, 0.05, 0.011, 0.25.$ Fit by Gompertz function with parameters $p_{1}=0.84\mu g/ml, p_{2}=3.34$. B) Probability distribution obtained from the data in panel A determined by numerical derivative, as described in Methods, MIC and MIA calculation part in main text, and from the fit presented in panel A. C) Comparison of probability distributions of individual MICs from panel B (red dashed line) and from experiments presented in Fig. 1 in the main text (black solid line). D) Same as C, but rescaled by their average single cell MICs defined by $c_{av}\equiv\int_{0}^{\infty} dc cp(c)$. Average single cell MIC for experiments in Fig 1, main text, is given by $c_{av}=0.29\mu g/ml$, whereas $c_{av}=0.65\mu g/ml$ for the data presented in panel A.


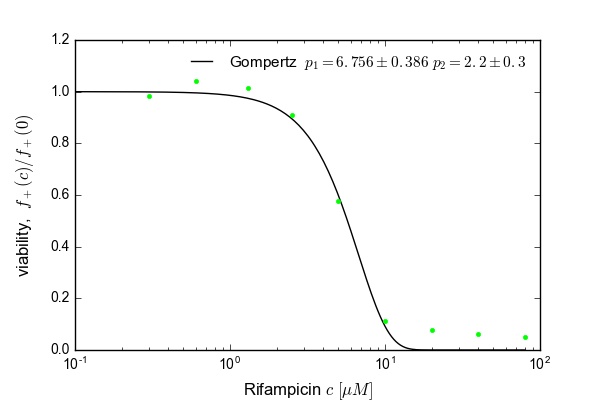

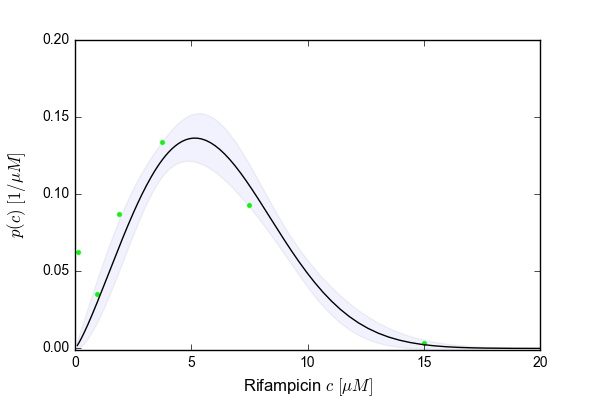


**Fig. S 7.** Eun et al.(7) determined fraction of positive droplets for encapsulation rate, λ=0.1. Their data presented in Fig. 3(7) allow to determine normalized fraction of positive droplets and probability distribution with the method described in this paper.


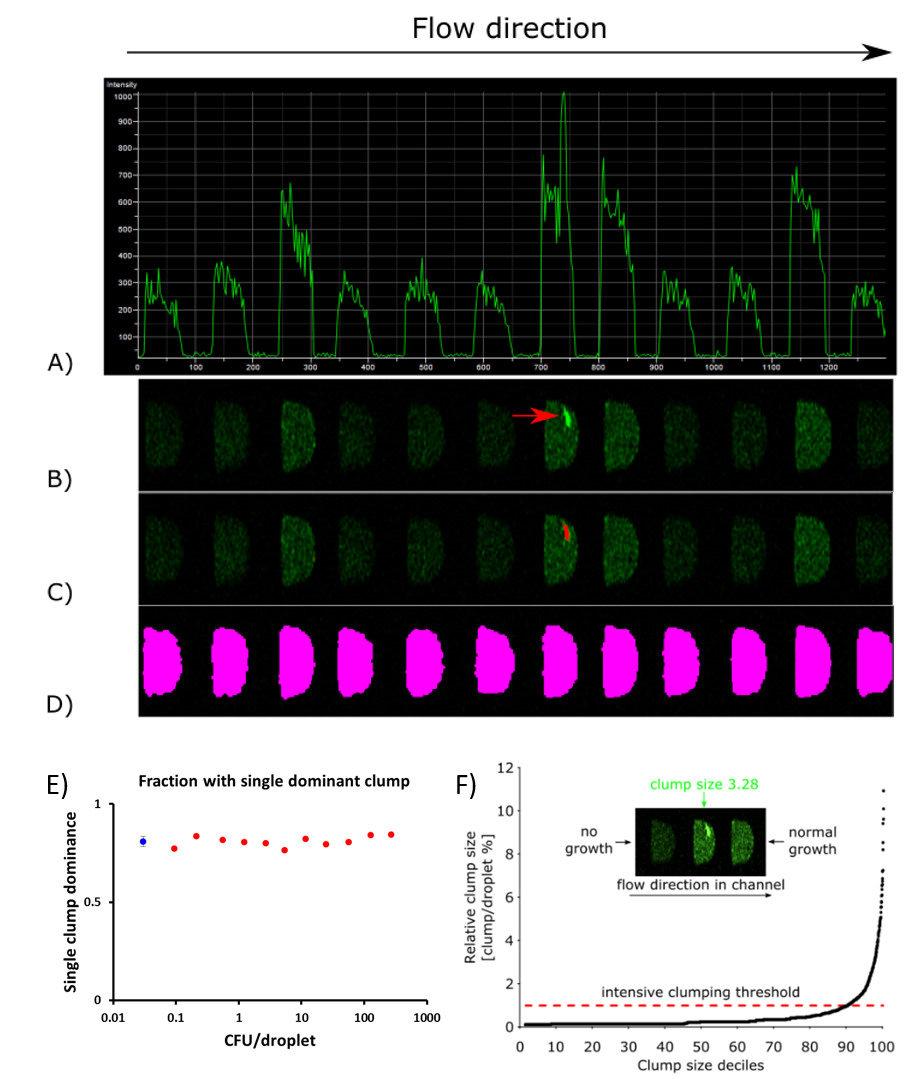


**Fig. S 8.** Bacteria clumping analysis. A)Example of droplet fluorescence signal track in confocal microscope. B) We observed clumping of bacteria manifested as clusters of high-intensity pixels in confocal line-scan stack (**red arrow**). First, we noticed clumping in droplets of control “virtual array” without the addition of antibiotics. To quantify the clumping events in our experiments we measured i) the existence of such high-intensity pixels in each droplet and ii) the relative size of each cluster if such event occurred in droplet. C) Clumping events were analyzed using object count function in Nikon NIS software with following settings. Thresholds: low (3000, threshold for high-intensity pixel) and high (4095, maximum signal), Smooth: OFF, Clean: OFF, Fill holes: ON, Separate: OFF. This measurement gives also shows the area of each clump (Ac). D) Relative size of clumps (S) was measured as area of clump (Ac) / area of droplet (Ad). Droplet sizes (Ad) were measured using binary function with following settings. Thresholds: low (100, minimum signal) and high (4095, maximum signal), Smooth: 4x, Clean: 2x, Fill holes: ON, Separate: OFF. We decided to measure clump size as relative to droplet size because the flow speed of droplets during imaging can fluctuate as the population of droplets pushed through the chip decreases. Such flow fluctuation may cause small changes in the apparent droplet cross-section area in the image. Clumping is not caused by debris or dust in the growth media as there were no clumping events detected at the beginning of experiment at 0h before the incubation. E) In case of clumping, the droplet is usually dominated by a single dominant clump. We observed that with ~80% of the clumping events the size of the biggest clump (A_c_) was more than half of the total area of measured clumps (A_sum_). The dominance of a single clump was similarly near 80% in all bacteria inoculum densities (**red dots** on the graph). **Blue dot** shows the average of red dots with respective standard deviation as error bars. F) The clumping was extremely intensive in certain fraction of droplets and this fraction is shown on the figure as top 90 decile of clump sizes in droplets. In our following analysis we use the 90th decile value (clump size ~1.06%) as a threshold for “intensive clumping”. The same analysis criteria were then used to measure clumping in virtual array experiments with different antibiotic concentrations.

**Table S 3.** Table showing the data about the percentage of droplets where bacteria demonstrated intensive clumping.

**Table S 4.** Table showing the average relative size of clumps in droplets, calculated as the percentage of droplet area. Relative size of clumps (S) was measured as area of clump (A_c_) / area of droplet (A_d_).

**References**

1. Li W, et al. (2008) Simultaneous generation of droplets with different dimensions in parallel integrated microfluidic droplet generators. Soft Matter 4(2):258.

2. Kaminski TS, Jakiela S, Czekalska MA, Postek W, Garstecki P (2012) Automated generation of libraries of nL droplets. Lab Chip 12:3995–4002.

3. Nie Z, et al. (2008) Emulsification in a microfluidic flow-focusing device: Effect of the viscosities of the liquids. Microfluid Nanofluidics 5:585–594.

4. Scheler O, et al. (2017) Optimized droplet digital CFU assay (ddCFU) provides precise quantification of bacteria over dynamic range of 6 logs and beyond. Lab Chip 17:1980–87.

5. Debski PR, Garstecki P (2016) Designing and interpretation of digital assays: Concentration of target in the sample and in the source of sample. Biomol Detect Quantif 10:24–30.

6. Lyu F, et al. (2018) Phenotyping antibiotic resistance with single-cell resolution for the detection of heteroresistance. Sensors Actuators, B Chem 270(February):396–404.

7. Taylor J (1997) Introduction to error analysis: The study of uncerntainties in physical measurements.
